# Unraveling the dynamics of ChatGPT adoption and utilization through Structural Equation Modeling

**DOI:** 10.1038/s41598-024-74406-4

**Published:** 2024-10-08

**Authors:** Khalida Parveen, Tran Quang Bao Phuc, Abdulelah A. Alghamdi, Fahima Hajjej, Waeal J. Obidallah, Yousef A. Alduraywish, Muhammad Shafiq

**Affiliations:** 1 School of Liberal Arts and Education, Shandong Xiehe University, Shandong, China; 2 Faculty of Foreign Languages, University of Foreign Languages and Information Technology, Ho Chi Minh City , Vietnam; 3 Department of Educational Policies, Faculty of Education, Umm al-Qura University, Makkah, Saudi Arabia; 4 College of Computer and Information Sciences, Princess Nourah bint Abdulrahman University, Riyadh, Saudi Arabia; 5 College of Computer and Information Sciences, Imam Mohammad Ibn Saud Islamic University (IMSIU), Riyadh, 11673 Saudi Arabia; 6 School of Information Engineering, Qujing Normal University, Yunnan, China

**Keywords:** Artificial intelligence, ChatGPT, Technology adoption, Behavioral intention, Higher education, Software, Information technology

## Abstract

ChatGPT, an advanced Artificial Intelligence tool, is getting considerable attention in higher education. ChatGPT significantly changes the student learning experience through its AI-aided support, personalized study assistance and effective educational experiences, and it has become an object of particular interest in this context. This research aimed to build a technology acceptance and usage model that encapsulates the elements influencing students’ adoption and utilization of ChatGPT, drawing on constructs from the ‘Unified Theory of Acceptance and Use of Technology’ and ‘Flow Theory’. The proposed model was found valid and prolific, with the credibility of the results relying on the self-reported surveys of 505 students from three universities in Pakistan. Structural Equation Modelling (SEM) was used to analyze data that confirmed the robustness and validity of the proposed model of the study. The study findings supported nine out of the ten proposed hypotheses. Perceived playfulness was declared the paramount predictor of behavioral intention, while perceived values and performance expectancy were the next-level predictors. Additionally, behavioral attention was a high and inspiring determinant of ChatGPT usage behavior, followed by attention focus. This analysis demonstrates a need for a thorough investigation of AI tools like ChatGPT in higher education.

## Introduction

In recent years, breakthroughs in artificial intelligence (AI) technology have resulted in tremendous progress in its widespread diffusion and exploitation^1,2^. Advances in AI have introduced advanced content-generation models that enable readers to rapidly create a wide range of digital media items and writing samples using basic text-based queries^3^. Consequently, there has been a significant surge in interest in new AI technologies^1^.

ChatGPT, Chat Generative Pre-trained Transformer-an OpenAI’s chatbot launched in 2022, is accessible to all by creating a free account of OpenAI^4,5^. In a very short time, ChatGPT has become popular among all fields as its professional artificial intelligence text generator created by OpenAI is quite versatile^2,4^. This AI language model produces original text in response to human instructions^4^. ChatGPT has become the fastest-growing commercial application in history^6,7^. Nevertheless, AI tools like ChatGPT have elevated disquiets in various industries and organizations^6,7^.

Since ChatGPT emerged, its features have garnered much attention from students and education professionals^2,6^. The ChatGPT system is being built using the GPT-3 family of models featuring substantial language models that have been fine-tuned for transfer learning^4,6^. These models are capable of utilizing both supervised and reinforced learning techniques. Technology integration in education settings, particularly the emergence of AI, has become a global concern for researchers in recent years^8^. Many scientific community members are discussing how to make ChatGPT work better in the classroom regarding its advantages, advancements, and ease^7,9^.

Numerous new and emerging topics are observed through a review of previous literature about ChatGPT usage in academia, particularly in higher education. Generally, these topics comprise the application of ChatGPT in normal schools and universities^10,11^. Let’s look at the use of ChatGPT in academics.

The adoption of ChatGPT in higher education literature courses represents a paradigm shift in pedagogy, harnessing the power of artificial intelligence to enhance literary analysis and scholarly inquiry^9,10^. Higher education has started benefiting from ChatGPT in terms of assessment and exploring how it supports learning^9^. Schools can use ChatGPT to help teachers evaluate assignments, develop students’ writing skills and critical thinking, and understand the relevance of AI tools in the contemporary world^8,10^.

ChatGPT is a powerful tool for creative learning, teaching, and evaluation consistent with a transformative approach to knowledge^9,10^. As highlighted in the works of^12,13^, ChatGPT catalyzes fostering intellectual curiosity and a deeper appreciation for literature’s cultural, historical, and social dimensions. Furthermore, the incorporation of ChatGPT in literature studies fosters a collaborative learning environment that transcends geographical boundaries and disciplinary silos^14,15^. By embracing ChatGPT as a partner in literary exploration, students enrich their academic experience and cultivate the skills and dispositions needed to navigate an increasingly complex and interconnected world^8,15^.

As referenced by recent studies^14,15^, integrating ChatGPT into the curriculum empowers students to explore texts in novel ways, leveraging its vast repository of knowledge and linguistic expertise. Using ChatGPT as a virtual tutor or discussion partner, students can receive personalized feedback, guidance, and supplementary materials tailored to their individual learning needs and preferences^15^. Drawing on the insights of scholars from diverse fields, students can engage in interdisciplinary discussions and research projects illuminating literary texts’ multifaceted nature and their relevance to contemporary issues^15^. This approach democratizes access to literary education and promotes active learning and critical thinking skills essential for academic success and lifelong learning^16^.

Various researchers described the effects of ChatGPT on the educational field^16,17^, though few have pointed to some of the advances in academia, like publishing and writing articles^18,19^and other wide-ranging areas of life. Nevertheless, as universities consider the implications of AI Chats software, many dedicated educators have already introduced it into their courseware to reveal its shortcomings and question its capabilities^19^. What future ChatGPT might have on higher education teaching and learning goals, as this technology is potentially very versatile^20^. The whole concept of applying ChatGPT in higher education and academia is still in its early stages^17,20^.

Due to the short duration of ChatGPT’s usage, there is a dearth of comprehensive empirical research and outcomes about its potential uses and advantages^21,22^. An evident study deficiency exists in the existing literature, which predominantly concentrates on the perspectives of academic educators and scientists about ChatGPT and its prospective development^21,22^. Due to the recent launch of the AI tool, there is currently insufficient understanding of how students perceive and use this new technology. We acknowledge that university students are essential stakeholders willing to integrate and utilize ChatGPT in their studies. Therefore, studies are needed to address students’ acceptance and adoption of ChatGPT.

In order to explore the acceptance of this technology, components of the already existing models, i.e., the Unified Theory of Acceptance and Use of Technology 1–2 (UTAUT1-2) formulated by^23,24^, and the flow theory conceptualized by Czikszentmihalyi^25^, i.e., Attention Focus, were employed. Indeed, the results of recent studies suggest the application of the models to evaluate new technologies in universities: for instance, the diffusion of mobile internet^26^, animation usage^27,28^, mobile devices for language learning^29^, E-learning in higher education during COVID-19^18,30^, or learning management system^31,32^. The theoretical concepts will shape a proposition in this paper that determines ChatGPT’s adoption and usage among university students. Therefore, the proposed model outlines seven factors that predict the usage and adoption of technology, including, performance expectancy effort expectancy, social influence, facilitating conditions, perceived value, perceived playfulness, and attention focus.

The study is structured in the following manner. The introduction section presents the first details regarding the ChatGPT advancements and the discussions regarding its application in higher education and academics. The hypothesis development delivers a comprehensive explanation of the proposed model’s constructs and the hypotheses developed to evaluate the adoption and usage of ChatGPT in university education. Additionally, the research methodology section features a customized assessment tool designed exclusively and launched to assess the model’s constructs. In the other section of the study, the outcomes of the SEM model using the partial least squares approach were explained, along with the estimation of the proposed theoretical model, followed by a discussion of study findings. Subsequently, the study’s originality and the objectives’ significance are highlighted.

## Hypotheses development

Understanding the factors that significantly influence students’ adoption and utilization of ChatGPT is essential for effective implementation and integration of this technology into educational practices. To this end, the proposed model integrates elements from the Unified Theory of Acceptance and Use of Technology 1–2 (UTAUT) formulated by^23,24^and Flow Theory conceptualized by^25^, capturing both extrinsic and intrinsic motivational significance in technology adoption relevant to the Pakistani context. The UTAUT 1–2 framework, widely recognized for its robustness in predicting technology adoption^18,24^, serves as the foundation for the proposed model. While UTAUT 1–2 mostly addresses extrinsic motivation, the Flow Theory complements it by incorporating intrinsic motivational factors, specifically perceived playfulness and attention focus^25^. The proposed model is particularly crafted for the context of Pakistan by considering the unique characteristics of Pakistani students, where technology adoption/utilization in education is significantly influenced by a combination of social, economic, and cultural factors. These constructs primarily reflect extrinsic and intrinsic motivational factors that drive technology usage, where users’ decisions for technology adoption and usage are driven by the expected benefits (Performance Expectancy) and ease of use (Effort Expectancy), as well as the influence of social factors (Social Influence), the availability of resources (Facilitating Conditions), perceived cost-benefit analysis (Perceived Value), the entertainment value (Perceived Playfulness), and cognitive engagement during use (Attention Focus) respectively. In the context of the current model, perceived value is not conceptualized in line with the price value from UTAUT, but rather considering the non-financial/satisfaction values associated with ChatGPT usage. The integration of UTAUT and Flow Theory provides a comprehensive understanding of the factors influencing students’ adoption and utilization of ChatGPT, bridging the gap between utilitarian and hedonic motivations.

Performance expectancy, as manifested by^23,33^, is the level of an individual’s expectations to use a specific technology to increase the effectiveness in accomplishing a given task or meeting its objectives. Performance expectancy is expressed as the user’s perceived utility gained from a specific technology, enabling the user to acquire information or service as well as possible anytime, anywhere, and significantly increasing their active and proactive performance and efficiency in life and work^24,26^.

As mentioned by^18^, performance expectancy is one of the most critical components that determine how well educational systems are received in academic settings. This is corroborated by the abundant scientific evidence showing a robust primary effect of performance expectancy on students’ behavioral intention in using innovative educational instruments^18,27,29^. For instance^34,35^, studied this metric concerning mobile learning and learning management systems, while^26,36^ used it in the case of Google Classroom. Hence, performance expectancy in this study relates to how likely students would think using ChatGPT would improve their productivity or academic performance. The following hypothesis (H1) is developed:

### Performance expectancy has a direct and significant impact on behavioral intention

Strzelecki^18^; Venkatesh, Morris^23^and Moore and Benbasat^37^define effort expectancy as the level of ease with which the user ensures that using a specific system can be done effort-free. The significant influence of effort expectancy on students’ behavioral intention to adopt different educational technologies has been brought to light by recent studies. For instance, research by Jakkaew and Hemrungrote^38^revealed that effort expectation plays a key role in adopting particular learning platforms such as Google Classroom. In a similar vein, Hu, Laxman^39^ noted the impact of effort expectancy with regard to mobile technologies in education. In a study, effort expectancy refers to how much users assume ChatGPT is easy to adopt and doesn’t require much work to adopt it. Here is the hypothesis (H2) given below:

### Effort expectancy has a direct and significant impact on behavioral intention

Social influence is previously described as the impact of the referees’ opinion on the behavior of an individual through a variety of terms^40^. Likewise, according to^23,41^, the degree to which a person believes that the persons who are significant to them believe they should utilize specific technological tools. More specifically, social influence theory proposes that people will be more likely to accede to the opinion of other central referees^42^. Therefore, when another central referee in a person’s life recommends that one use the mobile Internet, the person will accede to the recommendation^42^.

Social influence has a substantial impact on the behavioral intention of consumers to adopt technology in the classroom, which has been discussed in a number of studies^41,42^. The relationship between social influence and behavioral intention has been studied in various research with different contexts, including learning management systems^41^, use of ChatGPT^18^, and mobile learning^41,43^. Within the context of this research, the term social influence pertains to the extent to which students perceive that their instructors, peers, or other influential individuals in their social circle are endorsing or promoting ChatGPT. The subsequent hypothesis (H3) is put forth:

### Social influence has a direct and significant impact on behavioral intention

According to^23,44^, facilitating conditions is the extent to which a person believes the material, support, and resources exist and are approachable to use a specific technological tool efficiently. In addition to the convenience of ChatGPT training, technical assistance and students’ perceptions of their admittance to the technological tools, though these are highly demanding, are referred to as facilitating conditions^18^. Research has indicated that facilitating conditions is an important aspect in influencing a person’s usage of technological tools^40,44^, as they have been shown to be a key predictor for behavioral intention and ChatGPT user behavior. Furthermore, the adoption of a variety of educational technologies, including augmented reality technology in higher education^18,40^, and augmented reality^23,42^, has been found to depend critically on facilitating conditions. The following hypothesis (H4) is put forth:

### Facilitating conditions has a direct and significant impact on behavioral intention

Previous literature extensively supports the notion that facilitating conditions have a direct and significant impact on use behavior^18^. Facilitating conditions, which refer to the availability of resources, infrastructure, and support necessary for the effective use of technology, are crucial determinants of whether individuals will engage with and continue using a technological system^23,24,42^. Studies grounded in the Unified Theory of Acceptance and Use of Technology (UTAUT) consistently highlight the importance of facilitating conditions in influencing use behavior, particularly in environments where technological proficiency varies among users^35,39,40,43,44^. For instance, Venkatesh, Morris^23^found that the presence of adequate support systems, such as training programs, technical assistance, and accessible user interfaces, significantly enhances users’ confidence and willingness to adopt and sustain the use of new technologies. This effect is especially pronounced in contexts where the adoption of technology is not inherently intuitive or where users face challenges in integrating the technology into their daily routines^40,44^. Moreover, research by Teo^45^ emphasizes that facilitating conditions are not only pivotal in the initial adoption phase but also play a critical role in long-term usage, as ongoing access to resources and support ensures that users can overcome potential barriers to continued engagement. Thus, the consistent findings across various studies underline the critical role that facilitating conditions play in shaping and sustaining use behavior across different technological contexts. The following hypothesis (H5) is developed:

### Facilitating conditions has a direct and significant impact on use behavior

As a crucial construct in consumer behavior, the overall assessment and evaluation of the consumers regarding the usefulness or value of a service based on what the consumer receives and benefits is called perceived value^46^. Rooted in the perception of benefits versus sacrifices associated with a product or service, it intricately weaves into the fabric of consumer decision-making processes^46^. Moreover, perceived value holds way over behavioral intentions with paramount influence, likely fostering continuance usage^46^. As revealed in the seminal works of scholars like^46,47^, perceived value acts as a pivotal precursor to behavioral intentions concerning purchase, recommendation, or loyalty. This alignment between perceived value and behavioral intention elucidates the intrinsic motivation guiding consumer actions, underlining the significance of cultivating favorable perceptions among target audiences^26,48^. In this study, we propose to revise the conceptualization of perceived value to emphasize the valuable learning and interactive experience, given that the current access to ChatGPT is freely available to all users. In the case of university students using ChatGPT, the perceived value is not primarily rooted in financial cost but rather in the educational benefits and learning outcomes they derive from the interaction with the technology^18,32,41^. Accordingly, its value transcends traditional monetary considerations and instead centers on the intellectual and experiential gains ChatGPT users derive. Here, the perceived value is closely tied to the effectiveness of ChatGPT in enhancing their academic performance, supporting their learning processes, and contributing to their overall educational experience. This reconceptualization acknowledges that the ChatGPT platform serves as a distinctive and valuable resource for knowledge acquisition and interaction, thereby elevating its perceived value in purely non-financial terms. The following hypothesis (H6) is developed:

### Perceived value has a direct and significant impact on behavioral intention

Perceived playfulness, a crucial aspect of user experience in interactive systems, refers to the extent to which users find an activity enjoyable, fun, and intrinsically motivating^18,23^. This concept is rooted in Flow Theory, which posits that individuals are more likely to immerse themselves in activities that they find enjoyable and engaging^23^. Accordingly, perceived playfulness goes beyond utilitarian purposes and encompasses the degree of pleasure or fun derived from the interaction experience with a system^23,26^. When individuals perceive an interface or a product as playful, it enhances their motivation to engage with it. It fosters a positive attitude toward usage, ultimately leading to a greater inclination to adopt or continue using the system^24^. Perceived playfulness exerts a direct and noteworthy influence on behavioral intention, as supported by various studies e.g^49,50^. This relationship underscores the importance of incorporating playful elements into design strategies to captivate users and encourage sustained interaction and favorable behavioral outcomes^24,51^. Regarding the current study, perceived playfulness considers the extent to which students find using ChatGPT enjoyable and intrinsically satisfying, contributing to a more interactive and stimulating learning experience. The following hypothesis (H7) is developed.

### Perceived playfulness has a direct and significant impact on behavioral intention

Perceived playfulness has been widely recognized in the literature as a significant factor influencing use behavior, particularly in the context of technology adoption and continued usage^18,32^. Numerous studies have demonstrated that when users perceive a technology as playful, they are more likely to engage with it frequently and persistently^26,32^. Moon and Kim^49^found that perceived playfulness significantly enhances users’ behavioral intentions to use technology, as it creates a positive emotional experience that encourages repeated use. Similarly, research by Van der Heijden^51^suggests that perceived playfulness can reduce the cognitive load associated with learning new technologies, making the experience more enjoyable and less daunting, thereby leading to higher usage rates. The positive emotional response generated by playfulness also strengthens the user’s attachment to the technology, making it a key driver of sustained use behavior^25,51^. As such, perceived playfulness emerges as a crucial element in predicting and understanding how users interact with technology, particularly in environments where user engagement is critical for the success of the technology^51^. For instance, if their interaction with ChatGPT is perceived as enjoyable or engaging, users are more likely to move beyond initial exploration to consistent and meaningful use, regardless of the initial functional objective (e.g., answering a question, generating content, or assisting with tasks). Playfulness transforms the user experience from being purely task-oriented to one that feels exploratory and enjoyable, leading to stronger behavioral intentions and frequent use^18,51^. The following hypothesis (H8) is put forth:

### Perceived playfulness has a direct and significant impact on use behavior

Attention focus plays a pivotal role in shaping users’ behavior towards a product or system, exerting a direct and substantial influence on their utilization patterns^52,53^. When users are able to maintain focused attention on the features and functionalities of a product, they are more likely to engage with it actively and effectively, leading to increased usage^25,26^. This relationship underscores the importance of designing interfaces and experiences that facilitate users’ attentional engagement through clear visual cues, streamlined navigation, or compelling content to optimize user behavior and enhance overall user satisfaction and effectiveness^25^.

Attention focus assesses the degree to which ChatGPT captures and retains students’ attention, facilitating a deep state of engagement and flow during learning activities^16^. Attention focus encompasses the ability and willingness of students to creatively leverage this AI technology to enhance their learning experiences and academic pursuits^16^. It involves embracing ChatGPT as a versatile tool for conducting research, generating ideas, facilitating discussions, and even simulating tutoring or mentorship interactions^53^. Students with high attention focus in ChatGPT utilization are proactive in exploring its capabilities beyond conventional uses, experimenting with different prompts, refining their queries, and integrating generated responses into their coursework or projects.

By harnessing ChatGPT’s natural language processing abilities and vast knowledge base, students can engage in dynamic and intellectually stimulating exchanges, collaborating with AI to explore complex concepts, brainstorm solutions, and gain new perspectives^18^. This fosters a culture of innovation and intellectual curiosity within higher education, empowering students to transcend traditional boundaries and embrace AI as a valuable ally in their academic journey. The following hypothesis (H9) is developed:

### Attention focus has a direct and significant impact on behavioral intention

In the realm of technology acceptance theories, one of the most fundamental propositions at the heart of models like the Technology Acceptance Model and the Unified Theory of Acceptance and Use of Technology (UTAUT 1–2) is that behavioral intention directly influences use behavior^24,44,49^. According to the Committee on Communication for Behavior Change in the 21st Century, behavioral intention means subjective probability of a person to engage in a particular behavior^40^. Based on the research developed by^24,54^, behavioral intention is the subjective possibility of individuals using technology in the future. Particularly, behavioral intention refers to the user’s willingness or inclination to use a technology shaped by their attitudes towards its value or functionality, perceived usefulness, and perceived ease of use, etc^18,32,42^. The more experience consumers have, the more chances for innovativeness since they have more encounters with the cues and, thus, perform the behavior^33^.

Likewise, use behavior is also an important term when using any specific innovative technology^45^. The consistency and frequency of use depend heavily on the initial behavioral intention^52^. The stronger degree of a user’s intention to use a particular technology accelerates the likelihood of actual frequency and depth of usage behavior^54^. Once a strong behavioral intention is cemented, the next step in the technology acceptance process is transitioning to actual use behavior^40^. By ensuring that users have a useful, valuable, and playful experience with ease of use and efficiency as expected, their intention to use the tool will directly translate into sustained and frequent usage^18,41,44^. Interestingly, a positive and satisfactory experience is likely to create a feedback loop where actual usage further reinforces the intention to continue using the technology in the future^13,23,24^.

In the case of ChatGPT, as mentioned by^18,24^, this link refers to the utilitarian use of technology resources with the formation of behavior intention with respect to the frequency students plan to use ChatGPT during their postgraduate education. This involves integrating the tool into regular activities, i.e., using ChatGPT as a writing assistant or idea generator and leveraging ChatGPT for a variety of academic or research purposes, from personal assistance to professional tasks^16,18^. By focusing on exploring and improving factors that enhance behavioral intention, i.e., perceived playfulness, perceived value, a stronger link between intention and actual use behavior could be fostered. Ultimately, understanding the dynamics of this relationship is critical for driving students’ higher adoption rates and engagement with ChatGPT. The following hypothesis (H10) is developed:

### Behavioral intention has a direct and significant impact on use behavior

‘Study year’ and ‘Gender’ were added as moderating variables in this study. In order to keep the theoretical model simple - arising from the need to add a total sum of seven predictors and the short time in which the general public actively used ChatGPT. When researching students’ usage of ChatGPT, a moderator variable, like ‘Experience’ might be ignored because students often have similar levels of exposure to technology in an academic setting, leading to less variability in their experience. Additionally, the concept of applying ChatGPT in higher education and academia is still in its early stages.

**Fig. 1:**
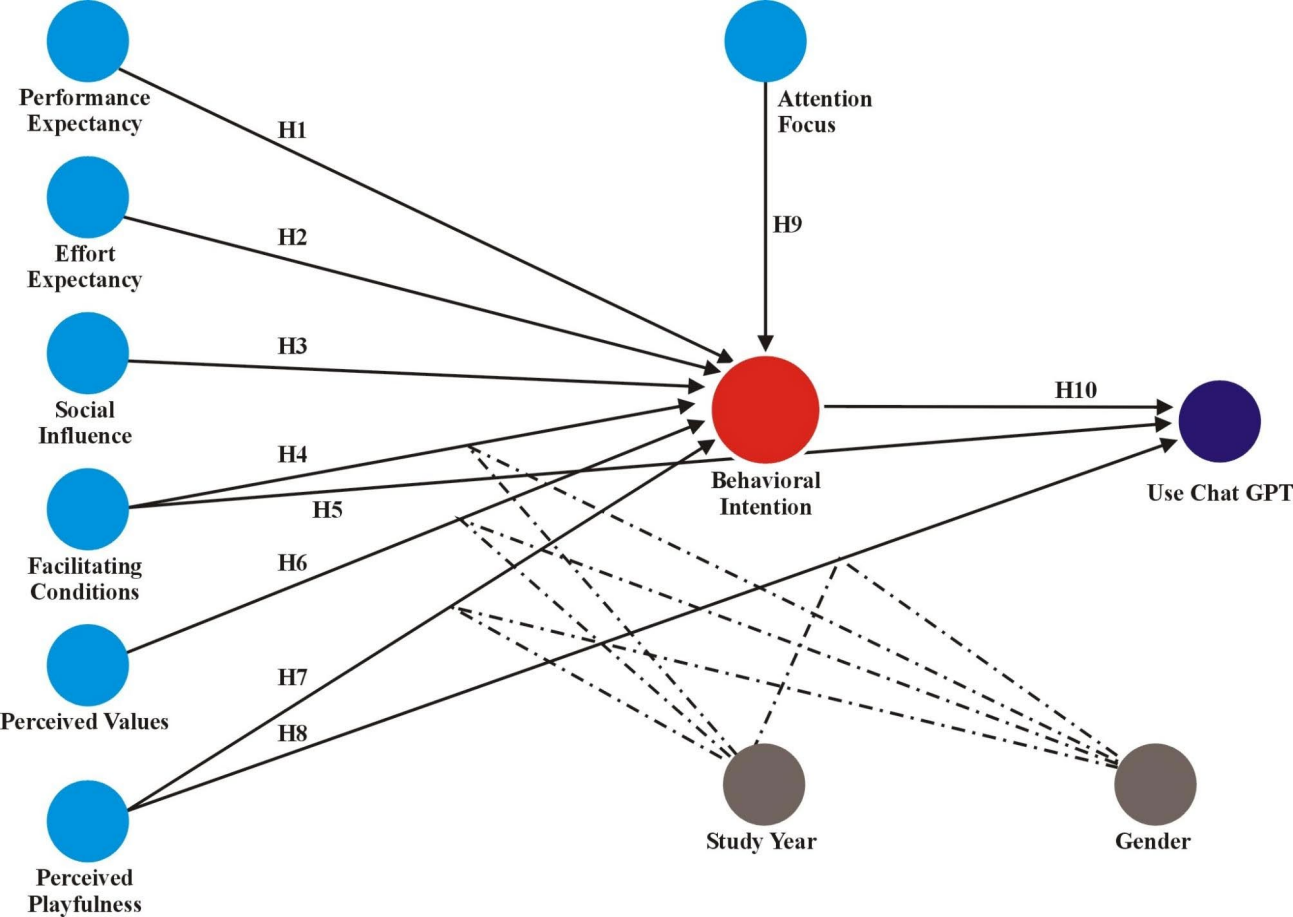
A proposed model for ChatGPT acceptance and usage.

The overall model is shown in Fig. 1, which presents the seven main predictors of the model, moderated by the study year and gender.

## Methodology

### Study measurement scale

A numerical metric scale with a range of 1 to 7 has been constructed for all exogenous constructs in the model to standardize the model estimation for every option. The majority of constructs were measured using a seven-point Likert scale, which ranged from ‘strongly disagree’ (1) to ‘strongly agree’ (7). This approach allowed for the capture of participants’ attitudes and perceptions across a continuum of agreement. We used thirty-five items in all modified from several studies by^18,23,24^. In adapting the constructs for a questionnaire aimed at assessing student acceptance of ChatGPT in Pakistan, several contextual factors were considered to ensure relevance and effectiveness. The constructs were tailored to address the unique educational environment and cultural context of Pakistan. The efficacy of the newly created scales was tested in pilot research involving 40 undergraduate students from GC University, Faisalabad (22 girls and 18 boys), prior to its distribution to the intended participants. Discriminant validity was confirmed, and each concept satisfied the reliability and validity requirements^55,56^. The use of ChatGPT was assessed in this study using a five-point rating system that went from ‘never’ to ‘once a day’. These responses were coded on a five-point scale where 1 corresponded to ‘never’, 2 to ‘once a month’, 3 to ‘once a week’, 4 to ‘several times a week’, and 5 to ‘once a day’, thus enabling the quantification of participants’ behavioral frequency. Table 1 provides a thorough display of the measuring scale and descriptive statistics.

**Table 1:** Main constructs, measurement scale, and factors.

Construct	Item	Details	Loading Factors	Mean	Standard Deviation	Adapted from the studies
Performance expectancy	PE1	Utilizing ChatGPT enhances your efficiency in academic pursuits	0.905	5.147	1.696	Strzeleck^18^
	PE2	ChatGPT expedites the completion of activities and projects in academic pursuits	0.870	4.702	1.789	
	PE3	Your chances of accomplishing significant academic goals are enhanced using ChatGPT	0.895	5.548	1.619	
	PE4	ChatGPT helps me in conceptual understanding of topics	0.867	4.873	1.857	
	PE5	ChatGPT helps solve problems	0.816	5.867	1.770	
	PE6	I think ChatGPT helps me with my homework assignments	0.803	4.398	1.675	
Effort expectancy	EE1	I find it effortless to acquire proficiency in utilizing ChatGPT	0.870	5.597	1.453	Venkatesh, Morris ^23^; Venkatesh, Thong ^24^
	EE2	I find ChatGPT user-friendly.	0.893	5.629	1.450	
	EE3	My interaction with ChatGPT is comprehensible	0.895	5.821	1.400	
	EE4	ChatGPT saves my time	0.912	5.801	1.523	
Social influence	SI1	Experts whose viewpoints are valuable to me recommend that I utilize ChatGPT	0.939	3.944	1.580	Strzelecki^18^
	SI2	The individuals who influence my behavior hold the belief that I ought to utilize ChatGPT	0.943	3.903	1.590	
	SI3	“My seniors advise me to use ChatGPT	0.937	3.897	1.629	
	SI4	Chat GPT helps me to shine among my peers	0.822	4.656	1.454	
Facilitating condition	FC1	I possess the requisite means to utilize ChatGPT	0.793	3.798	1.498	Strzelecki^18^; Venkatesh, Thong ^24^
	FC2	I can seek assistance from others when I encounter challenges while using ChatGPT	0.912	3.867	1.487	
	FC3	ChatGPT is well-suited for me compared with other technologies I utilize	0.795	3.985	1.731	
	FC4	I possess the requisite knowledge to utilize ChatGPT	0.790	3.944	1.580	
Perceived value	PV1	I feel motivated using ChatGPT	0.955	5.819	1.537	Shoufan^16^; Venkatesh, Thong ^24^
	PV2	ChatGPT provides a good explanation	0.962	5.756	1.505	
	PV3	ChatGPT provides authentic explanation	0.729	5.210	1.716	
	PV4	ChatGPT answers are well-structured	0.866	5.313	1.665	
	PV5	It’s a complimentary learning source	0.819	5.412	1.463	
Perceived playfulness	PP1	ChatGPT is better than other search engines	0.893	5.819	1.537	Ain, Kaur^41^
	PP2	ChatGPT makes a human-like, friendly impression	0.846	5.756	1.505	
	PP3	I feel good impact of ChatGPT usage on me	0.783	5.210	1.716	
	PP4	I feel confident using ChatGPT	0.890	5.819	1.537	
Attention focus	AF1	ChatGPT maintains a coherent thread of discussion	0.905	5.371	1.789	Shoufan^16^; Venkatesh, Thong ^24^
	AF2	ChatGPT has increased my level of focus	0.832	3.383	1.912	
	AF3	ChatGPT is a prompt source for me	0.939	4.433	2.048	
	AF4	I feel attentive using ChatGPT	0.914	5.752	1.591	
Behavioral Intention	BI1	I plan to keep utilizing ChatGPT in the future	0.910	5.379	1.667	Strzeleck^18^
	BI2	I intend to make consistent use of ChatGPT in my studies	0.823	5.363	1.784	
	BI3	I intend to keep using ChatGPT regularly	0.786	5.288	1.767	
Use ChatGPT	UC1	Please choose frequency according to the usage of ChatGPT: “Never”, “Once a month”, “Once a week”, “Several times a week”, and " once a day”	1.000	3.435	1.597	Venkatesh, Thong ^24^

### Sample characteristics

Selecting the appropriate sample size for a Partial Least Squares Structural Equation Modelling (PLS-SEM) is essential to guarantee the validity and accuracy of the results^55^. The complexity of the model, the number of latent variables and indicators, the magnitudes of the effects, and the required degree of statistical power are some of the factors that affect the sample size in PLS-SEM investigations, which are not fixed^56^. Some researchers suggest a minimum sample size of 100–200 observations. Various researchers have advised that a 5:1 or 10:1 ratio for sample size should be used^55,57^. Because thirty-five indicators are used for this study, about 300 sample size of participants is necessary^56^ (Table 1).

**Fig. 2:**
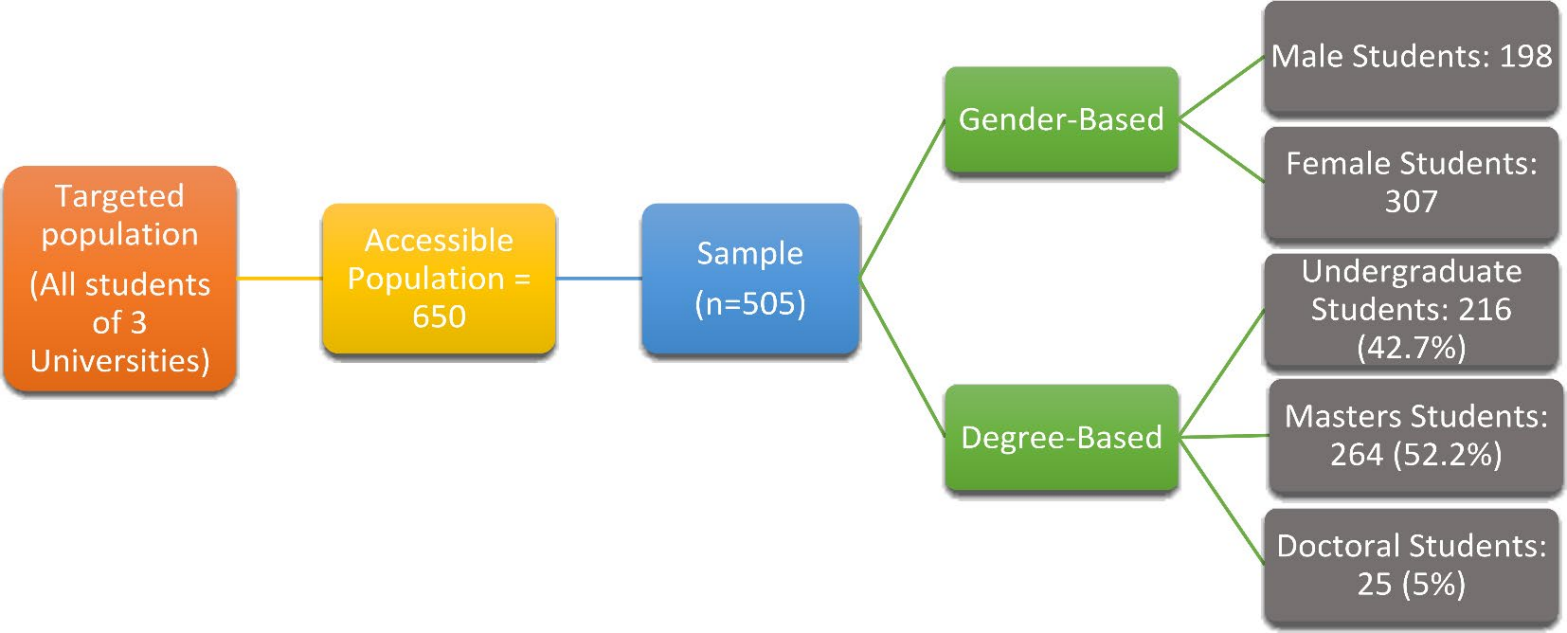
Demographics of Sample.

In early January 2023, the survey was conducted by distributing questionnaires to 650 students of various departments of three public universities in Pakistan, i.e., GC University Faisalabad, GCW University Faisalabad, and University of Agriculture Faisalabad. 650 questionnaires were sent to the students of all three universities by using simple random sampling, out of them 505 valid responses were received. There were 307 female students (60.7%) and 198 male students (39.2%) in the sample. With 216 students (42.7%) from bachelor’s degree programs, 25 (5%) PhD candidates and 264 students (52.2%) master’s degree program were included in the sample (Fig. 2). Students are chosen for ChatGPT usage research because they represent a key demographic in adopting and impacting emerging educational technologies. Selecting university students as participants in a ChatGPT study holds significant relevance due to their familiarity with technology, active engagement in digital communication, and adaptability to new tools^5,58^. Students, as digital natives, are often early adopters of new technologies and are an ideal population for studying the integration of AI. The wide range of academic tasks they usually have, from essay writing to problem-solving, offers a rich data set to determine where ChatGPT works and fails. Additionally, they are accustomed to research participation, providing reliable data for behavioral studies^12,23^. Moreover, conducting research on general participants, rather than solely on students, for ChatGPT usage could yield different results due to the varied demographics, backgrounds, and purposes of use among the broader population. General participants might include professionals, hobbyists, or individuals with specific needs, leading to more diverse usage patterns, motivations, and levels of technological proficiency. Unlike students, who may primarily use ChatGPT for academic purposes, general participants might utilize it for a wider range of activities, such as creative writing, professional tasks, or casual information-seeking. These differences could result in varied outcomes in terms of engagement, satisfaction, and perceived value of the tool, highlighting the importance of considering the diversity of user experiences in research studies^27^.

## Findings

The PLS-SEM analysis approach involves two primary steps: the measurement model assessment and the structural model assessment^56^. The measurement model is concerned with a thorough assessment of reliability and validity, i.e., factor loading composite reliability (CR), Cronbach’s alpha (CA), reliability coefficient (RC), average variance extracted (AVE), and discriminant validity^56,57^. Once the measurement model has been validated, the next step is to evaluate the structural model, focusing on evaluating the strength and significance of the path coefficients, and the explanatory power of the model, i.e., coefficient of determination (R²) and effect size (*f*²)^56,57^.

The SmartPLS 4 software was utilized, and the model was estimated using the PLS-SEM algorithm, with up to 3000 iterations and default initial weights^59^. Further^18,56^, recommended the use of bootstrapping, a nonparametric procedure with a single run of 5000 samples to test the statistical significance of PLS-SEM outcomes. The construct can be considered reliable when the factor loading of the indicator is 0.7 or greater, at that point where more than 50% of the variance in the indicator is explained by the construct^60^. An analysis of the construct was conducted keeping in view the use of the indicator loadings^60^. As all the 35 items have a loading factor higher than 0.7 (Table 1) therefore, the model’s 35 items were used to check the model.

According to Sarstedt, Ringle^60^, composite reliability is a criterion used to assess dependability. Values between 0.70 and 0.95 signify good and acceptable reliability levels. In order to test the internal consistency of the predictors, Cronbach’s alpha was employed to check the comparable thresholds of composite reliability^60^. A different reliability coefficient, derived from^61^, was also employed to offer a precise and uniform substitute. The convergent validity of the measurement models was evaluated by calculating the average variance extracted (AVE) from all items associated with a particular reflective variable^60^. A criterion of 0.50 or more for AVE was considered satisfactory^18,59,62^. The quality requirements listed in Table 2 were satisfied by Cronbach’s alpha, composite reliability, AVE, and reliability coefficient.

**Table 2:** Constructing the reliability and convergent validity.

Constructs	CA	RC	CR	AVE
Performance expectancy	0.918	0.923	0.915	0.759
Effort expectancy	0.887	0.868	0.884	0.843
Social influence	0.905	0.920	0.921	0.756
Facilitating conditions	0.925	0.891	0.878	0.809
Perceived value	0.968	0.977	0.946	0.788
Perceived playfulness	0.834	0.867	0.899	0.741
Attention focus	0.960	0.984	0.943	0.769
Behavioral Intention	0.823	0.845	0.946	0.856

*(CA*) = Cronbach’s Alpha, (*RC*) = Reliability Coefficient, (*CR*) = Composite Reliability, AVE = Average Variance Extracted.

The convergent validity and reliability of the individual scales and constructs have been satisfied. Additionally, the discriminant validity has been assessed, as shown in Table 3, where the square roots of the AVE values exceed the correlations between the constructs.

**Table 3:** Discriminant validity - Fornell-Larcker criterion.

Construct	BI	EE	FC	PV	PP	AF	PE	SI	UC
BI	0.674								
EE	0.762	0.698							
FC	0.701	0.768	0.653						
PV	0.567	0.694	0.798	0.756					
PP	0.655	0.712	0.698	0.785	0.825				
AF	0.632	0.576	0.857	0.654	0.734	0.456			
PE	0.705	0.791	0.689	0.748	0.509	0.656	0.498		
SI	0.689	0.789	0.702	0.658	0.687	0.377	0.435	0.655	
UC	0.589	0.687	0.657	0.740	0.552	0.577	0.707	0.697	0.802

According to Hair Jr, Hair Jr^62^established the heterotrait-monotrait ratio of correlations (HTMT), which is the recommended method for analyzing discriminant validity in PLS-SEM. When concepts are practically similar, an HTMT threshold of 0.90 is advised to assure discriminant validity; for more dissimilar constructs, a threshold of 0.85 is more suitable^62^. Every value in Table 4 is below the 0.85 cut-off, demonstrating strong discriminant validity.

**Table 4:** Heterotrait–Monotrait ratio of correlations.

Construct	BI	EE	FC	PV	PP	AF	PE	SI	UC
BI									
EE	0.767								
FC	0.687	0.596							
PV	0.736	0.761	0.522						
PP	0.867	0.696	0.377	0.656					
AF	0.751	0.745	0.587	0.456	0.656				
PE	0.767	0.467	0.456	0.339	0.734	0.456			
SI	0.711	0.508	0.766	0.688	0.567	0.656	0.498		
UC	0.652	0.666	0.405	0.546	0.845	0.377	0.435	0.655	

The entire model and strength of all constructs are evaluated by calculating the coefficient of determination (*R*^*2*^)^62^. Higher values of *R*^*2*^, which go from 0 to 1, suggest a more remarkable explanatory ability. As per the view of^60,63^, *R*^*2*^ values of 0.25, 0.50, and 0.75 are generally regarded as weak, moderate, and considerable, respectively. Moreover, *f*^*2*^values of 0.02, 0.15, and 0.35 indicate high, medium, and minor effects, respectively, whereas values less than 0.02 imply no effect^60^. These values are used to calculate the effect size of a variable.

**Fig. 3:**
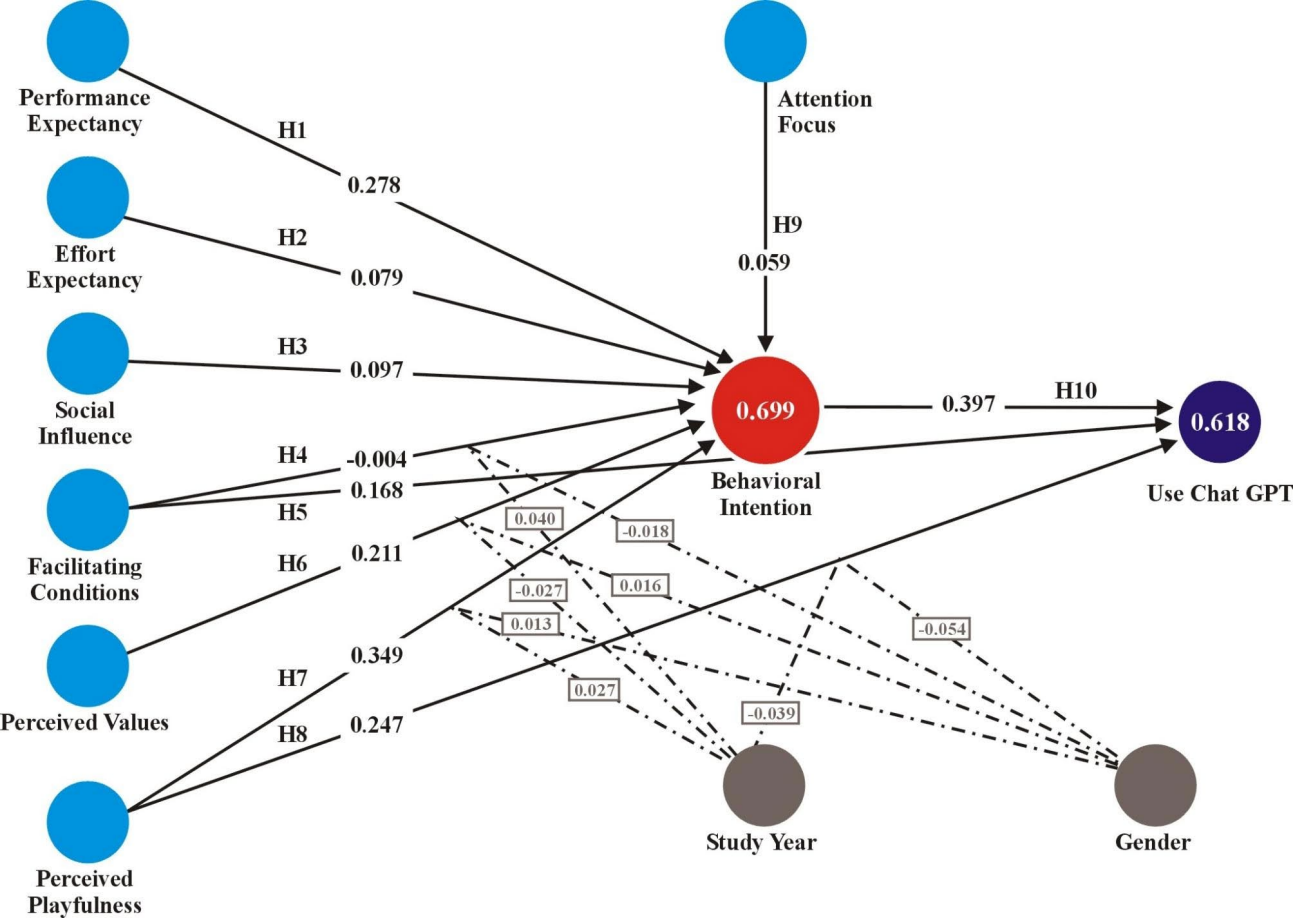
The outcomes for acceptance and usage of ChatGPT.

Figure 3 demonstrates the findings of PLS-SEM analysis, where the correlations between the variables are shown by the standardized regression coefficients, and the *R*^*2*^ values are displayed. The results of the research showed that perceived playfulness, with a coefficient of 0.349, was declared as the best predictor of behavioral intention, followed by performance expectancy (0.278) and perceived values (0.211). Additionally, positive impacts on behavioral intention were also noted for social influence (0.097), effort expectancy (0.079), and attention focus (0.059) though these connections did not have a significant *f*^2^ effect size. The constructs together explained 69.9% of the variation in behavioral intention. H4 is the only hypothesis that did not receive any support since the impact of facilitating conditions on behavioral intention (-0.004) was not demonstrated. ‘Facilitating conditions’ such as access to technology, support systems, and infrastructure, may not be as critical as other factors like perceived usefulness and ease of use. Despite potential limitations in facilitating conditions, students might still adopt ChatGPT due to its practical benefits in learning, ease of access, and widespread availability on various devices. This suggests that the value and convenience offered by the tool can outweigh the challenges posed by less-than-ideal supporting conditions, making it a viable option for students regardless of these external factors.

Regarding the impacts of behavioral intention, perceived playfulness, and facilitating conditions on ChatGPT use behavior, behavioral intention (0.397) had the greatest influence on Use ChatGPT, with perceived playfulness (0.247) and facilitating conditions (0.168) observed very close. Collectively, these three factors explained 61.8% of the variation in ChatGPT use behavior. Table 5 presents the path coefficients’ significance and confirmation of the hypothesis of the structural model.

**Table 5:** Coefficients of paths and their significance tests.

	Path	Coefficient	*P*-Values	f2	Confirmed / Not confirmed
Hypothesis1	PE ◊ BI	0.278	0.000	0.101	Confirmed
Hypothesis 2	EE ◊ BI	0.079	0.029	0.021	Confirmed
Hypothesis 3	SI◊ BI	0.097	0.001	0.019	Confirmed
Hypothesis 4	FC ◊ BI	-0.004	0.905	0.000	Not confirmed
Hypothesis 5	FC ◊ UC	0.168	0.000	0.047	Confirmed
Hypothesis 6	PV ◊ BI	0.211	0.000	0.058	Confirmed
Hypothesis 7	PP◊ BI	0.349	0.000	0.267	Confirmed
Hypothesis 8	PP◊ UC	0.247	0.000	0.069	Confirmed
Hypothesis 9	AF◊ BI	0.059	0.019	0.009	Confirmed
Hypothesis 10	BI◊ UC	0.397	0.000	0.159	Confirmed

The moderating associations between ‘Gender’ and ‘Study year’, expressly investigated and postulated a priori, have been integrated into the model. The results show that the associations between predictors and dependent variables that were investigated were not significantly affected by either of the two moderating variables. Table 6 displays the outcomes of the moderating effects of ‘Gender’ and ‘Study year.

**Table 6:** Paths and their moderating effects.

Variable Path	Coefficient	*P* Values	f2	Confirmation
Study year x PP ◊ BI	0.027	0.189	0.005	No
Study year x PP ◊ UC	-0.039	0.186	0.003	No
Study year x FC◊ BI	0.040	0.308	0.005	No
Study year x PV◊ BI	-0.027	0.178	0.003	No
Gender x PP◊ BI	0.013	0.709	0.000	No
Gender x PP◊ UC	-0.054	0.388	0.006	No
Gender x PV◊ BI	0.016	0.809	0.000	No
Gender x FC◊ BI	-0.018	0.756	0.002	No

Note: Statistically insignificant due to *P*-value > 0.05.

## Discussion

We assessed the acceptability and use of ChatGPT using the key constructs of the Unified Theory of Acceptance and Use of Technology 1–2 (UTAUT1-2) formulated by^23,24^, and the flow theory conceptualized by Czikszentmihalyi^25^, and all seven external variables satisfied the reliability as well as validity criteria. Regarding the impacts of all seven exogenous constructs on behavioral intention, according to our findings, three variables, i.e., performance expectancy, perceived value and perceived playfulness, are positively correlated with behavioral intention. The findings are consistent with studies by Arain, Hussain^35^about students’ intention/acceptance of mobile learning in the context of higher education in Pakistan, Strzelecki^18^on Polish students’ intention/adoption and use of ChatGPT, Twum, Ofori^64^investigating students’ intention to use E-learning during the COVID-19 pandemic, and Zwain^31^ featuring faculty and students intention/acceptance of the Moodle-Learning Management System in Iraq.

Perceived playfulness was declared the paramount predictor of behavioral intention, while perceived values and performance expectancy were the next-level predictors. The bulk of research on technology acceptability in higher education has also discovered a strong positive correlation between perceived playfulness and behavioral intention^18,31,35,64,65^. Accordingly, research indicates that perceived playfulness has a significant positive impact on faculty and students’ acceptance of the Moodle-Learning Management System^31^, Sub-Saharan Africa/Ghana students adoption of e-learning in response to COVID-19^42^, or medical students’ behavioral intention to use blended learning^65^. However, our result is in contrast to those of^66,67^ who demonstrated that perceived playfulness did not show any direct association with behavioral intention regarding EFL graduate Yemen students’ behavioral intention to use Google Classroom platform and the Gambia and UK workers adoption of e-learning, respectively.

Based on our research, performance expectancy is the second-best predictor of behavioral intention. Similar results are presented in previous studies that behavioral intention’ has positive connection with performance expectancy in adopting and utilizing emerging technologies across multiple contexts^18,31,35,64,65,68^. Accordingly, performance expectancy has emerged as a valid predictor of behavioral intention in developing students’ attitudes toward utilizing video conferencing tools for learning from the perspective of Ghana university students for a blended course during the COVID-19 pandemic^69^. Likewise, this finding is in agreement with previous findings, i.e., students’ behavioral intention to use animation and storytelling applying the UTAUT model^70^, e-learning system studies^71^, the intention to use interactive whiteboards in classrooms^72^, or e-learning system by state university students in Sri Lanka^73^. Nonetheless, this finding is contradictory to Alotumi^66^study indicating that performance expectancy had no direct effect on behavioral intention. As a result of the present study, a positive correlation between behavioral intention and perceived values was found in utilizing ChatGPT. Previous research on introducing emerging technologies in the educational field, like faculty and students intention/acceptance of the Moodle-Learning Management System in Iraq^31^and Malaysian university students intention towards learning management system with respect to the influence of learning value^41^, also provided the same findings.

Our study concludes that though effort expectancy and social influence have a statistically beneficial impact on behavioral intention, their *f*^2^values are less than 0.02. As calculated using students’ responses, effort expectancy reached the highest mean values among all variables and proved that ChatGPT is widely used and acceptable by the students. It indicates that applying this technology in higher education requires little effort and has no impact on behavioral intention. Students are fast learners and early adopters, so they usually find new technologies easy to utilize^36^. Research on students who use Microsoft PowerPoint in higher education and on e-learning platforms demonstrated similar results^67,74^. In contrast, effort expectancy has no statistically significant impact on the Pakistani students’ behavioral intention toward mobile-learning acceptance as investigated by Arain, Hussain^35^.

Several studies showed that social influence has an effect on behavioral intention in earlier-adopted technology, such as mobile learning^75^, e-learning system by state university students in Sri Lanka^73^. However, few studies revealed that social influence has no statistically significant influence on the students’ behavioral intention toward technology acceptance, i.e., Google Classroom^66^, mobile learning^35^, or Moodle-Learning Management System^31^. The result of our study showed that social influence has zero influence on behavioral intention to use ChatGPT. As a result, the ChatGPT conversation is more likely to be used by early adopters and experts in their field; they are not affected by any outer source. It is explicit that there is no societal pressure to use ChatGPT, as it has not yet reached mass implementation and adoption. Also, when universities create recommendations with respect to using ChatGPT and other technology tools, then social influence might get significance^36,66^. In collectivist cultures like Pakistan, where social norms and group behavior significantly shape individual actions, the endorsement of ChatGPT by influential figures within academic circles could accelerate its acceptance.

The variable attention focus was observed to have a small positive impact on behavioral intention, with *f*^2^value of less than 0.02. This finding is similar to a study by Alwahaishi and Snášel^26^that empirically investigated respondents in Saudi Arabia regarding the acceptance and use of Mobile Internet. This outcome implies that students may possess a restricted level of familiarity with ChatGPT and may lack sufficient experience in utilizing it. As per the findings of the present study, facilitating conditions did not show any statistical significance towards behavioral intention. This finding is in line with the findings of^66^Google Classroom acceptance by EFL graduate students^35^, Pakistani students’ intention/acceptance of mobile learning^18^, Polish students adoption and use of ChatGPT, or^68^patients to use a mobile health education website. Nevertheless, facilitating conditions had a substantial influence on ChatGPT use behavior as demonstrated in the model paradigm. This finding is in agreement with previous studies by^18,31,41,73^. The utilized model accounts for 69.9% of the variability in behavioral intention, demonstrating a significant level of descriptive capability. This finding underscores the importance of strengthening behavioral intentions to drive actual technology use. Moreover, the concepts of behavioral intention, perceived playfulness, and facilitating conditions exert a substantial and immediate influence on ChatGPT use behavior, as elucidated by the model with a moderate degree of 61.8%.

The proposed technology acceptance model integrating the Unified Theory of Acceptance and Use of Technology with Flow Theory aims to provide a comprehensive understanding of both extrinsic and intrinsic motivational factors that influence students’ adoption and utilization of ChatGPT in the Pakistani context. It is explicit from the study findings that this is particularly relevant in the educational context, where students may engage with ChatGPT not only for academic purposes but also for exploration and learning in a more interactive and enjoyable manner^8,18^. A playful interaction can enhance students’ engagement, leading to more frequent and prolonged use of the technology^35,64^. Given the increasing reliance on AI-driven tools for educational purposes, it is anticipated that students will adopt ChatGPT if they perceive it as a means to improve learning outcomes, streamline academic tasks, and increase productivity. Moreover, in the context of university students, particularly in Pakistan where digital literacy varies significantly, the perceived ease of interacting with ChatGPT will likely play a crucial role in its adoption. According to our work, the use of the dialogue interface of ChatGPT that attracts users and permits a wide variety of interactions among the factors provided by students could be pleasant and interesting. Our findings suggest students with high performance expectancy are more likely to adopt useful technology like ChatGPT. Students are at ease embracing new technologies and that frequent usage helps shape behavior, particularly regarding AI-powered chat services like ChatGPT^16^. The research indicates a user-friendly behavior regarding the use and adoption of ChatGPT. To utilize it, no other resources or devices are required, and it functions autonomously.

Additionally, the PLS-SEM results indicate that effort expectancy, social influence, and attention focus have an influence on behavioral intention, but with *f*^2^values less than 0.02, their effect sizes are minimal. The low effect size of effort expectancy might suggest that students do not perceive ChatGPT as particularly challenging to use, thus diminishing its importance as a predictor. The minimal impact of social influence could indicate that students’ decisions to adopt ChatGPT are more individually driven rather than influenced by peers or societal expectations. The study also uncovers an intriguing non-significant finding, particularly concerning facilitating conditions, which did not significantly influence behavioral intention. The lack of a significant relationship may reflect the increasing accessibility of technology among university students in Pakistan, particularly in urbanized areas, who might already have the necessary resources to use ChatGPT effectively^35^. This could include widespread availability of internet access, familiarity with digital tools, and institutional support for using AI in education. Alternatively, students may not perceive facilitating conditions as a barrier, given that ChatGPT is a web-based tool requiring minimal technical setup, reducing the perceived importance of facilitating conditions. Another possible explanation could be that university students in Pakistan may not perceive external resources and support as necessary for using ChatGPT, especially if they consider themselves already proficient with technology. This suggests that the traditional emphasis on facilitating conditions might be less relevant for younger, more technologically literate populations.

## Study conclusion and limitations

In the context of Pakistan, where higher education institutions are increasingly adopting digital tools to enhance learning outcomes^35^, understanding the factors that drive students’ acceptance of technologies like ChatGPT is crucial. By integrating both extrinsic and intrinsic motivational factors, this model offers a holistic approach to predicting and enhancing the adoption of ChatGPT among university students in Pakistan.

This study sought to give a clear picture of students’ perception of usage and adoption of ChatGPT, in addition to validating the strong influence of perceived playfulness, performance expectancy, and perceived values on behavioral intention to use ChatGPT. This study stands out because to its focus on ChatGPT and a newly established proposed model that still needs to be examined in the setting of higher education. There are only a few previous researches on ChatGPT^15–18^, particularly in relation to its utilization and reception in higher education settings, which underscores the originality of the present study. Hence, the study outcomes could significantly enhance the comprehension of ChatGPT’s acceptance and usage in higher education, as well as aid in the foundation of efficient applications of ChatGPT in higher educational settings.

Nevertheless, this study is constrained with the limitation that data was only obtained from three universities of Pakistan although with a wide representation of students’ academic backgrounds. Given that the utilization of ChatGPT in higher education is still a developing field of study, the next research might assess and enhance the scale applied in this study in different contexts for future investigations.

## Study implications

Our study contributes to the present comprehension of students’ perceptions of ChatGPT. Despite the paucity of research on the subject, especially in the context of higher education, our findings have significant ramifications for advancing the discussion on the application of AI chat technology as a teaching tool. Gathering such information will have a far-reaching impact on the scope of education and technology, mainly due to its usefulness in understanding how to incorporate new tools and technologies into the education sector to improve student learning. Furthermore, pinpointing the predictors influencing usage patterns will enable teachers and developers to provide targeted enhanced support and user experience, confirming that ChatGPT most accurately represents students’ preferences and needs.

The findings of this study offer significant implications for various stakeholders in the educational ecosystem, particularly in the realms of educational technology development, policy-making, and curriculum design. Accordingly, the finding that perceived playfulness is found to be the primary predictor of adoption indicates a need for educational technology developers to focus on creating AI tools that are not only functional but also engaging and enjoyable for students. This could involve incorporating gamified elements, interactive features, and user-friendly interfaces that enhance the overall user experience. By emphasizing playfulness, the likelihood of student adoption and sustained use of AI tools in educational contexts can be increased. For curriculum designers, these findings suggest a need for thoughtful integration of AI tools like ChatGPT into the teaching and learning process in ways that enhance both the perceived enjoyment and the educational value of the learning experience.

Therefore, cultural influences, ethical considerations, and consequences on learning outcomes and feedback need to be explored responsibly and effectively. It will further help produce more knowledgeable researchers who are willing to explore AI-driven technologies in higher education.

## Recommendations

Several recommendations could be derived from the findings of the study focusing on students’ acceptance and use of technology, such as ChatGPT, to ensure its proper integration into educational environments. Policymakers are responsible for creating an enabling environment for the integration of AI in education. The findings suggest that while students are generally positive about the use of AI, there is a need for clear guidelines and policies to govern its use in academia. Leadership programs should be introduced to familiarize local educational leaders with the potential and limitations of AI tools like ChatGPT, enabling them to make informed decisions regarding their adoption and usage. Continuous training and the establishment of support systems should also be provided to educators to enable improved teaching practices. A comprehensive system of monitoring and evaluation of students’ experiences and learning results should be implemented to allow iterative improvements and ensure that ChatGPT and tools like that meet educational objectives and student needs.

From the perspective of students, the ethical use of ChatGPT requires a deep understanding of the potential consequences of misuse, such as academic dishonesty and the devaluation of critical thinking skills. Ensuring that students are adequately informed about the benefits and ethical concerns associated with the use of AI-driven tools should be one of the primary ongoing activities, i.e., training sessions and workshops to sensitize students to the ethical implications of ChatGPT, emphasizing the importance of originality in academic work. In addition, improvements in the area of user-centered design will significantly impact usability and accessibility.

Several areas warrant further exploration to address the limitations and gaps identified in the current research. This study was limited to students from three universities in Pakistan, which may not fully represent the diverse educational landscape of the country. Future research should expand the sample to include a broader range of universities, including those from different regions, private and public institutions, and varying academic disciplines. To capture the evolving nature of students’ attitudes and behaviors towards ChatGPT, longitudinal studies are recommended. Such research would allow for tracking changes over time, particularly as students become more familiar with the technology and as ChatGPT undergoes updates. The exclusive use of a quantitative approach may limit the depth of understanding regarding students’ perspectives. The inclusion of qualitative data, such as interviews or focus group discussions, might result in a complete understanding of the complexities surrounding the adoption and usage of ChatGPT among university students. The non-significance of facilitating conditions and the minimal effect sizes of effort expectancy, social influence, and attention focus highlight the need for a deeper examination of these constructs in future research.

## Data Availability

The raw data supporting the conclusions of this article will be available by the corresponding author without undue reservation.
