# Author Correction: Unraveling the dynamics of ChatGPT adoption and utilization through Structural Equation Modeling

**DOI:** 10.1038/s41598-024-79470-4

**Published:** 2024-11-15

**Authors:** Khalida Parveen, Tran Quang Bao Phuc, Abdulelah A. Alghamdi, Fahima Hajjej, Waeal J. Obidallah, Yousef A. Alduraywish, Muhammad Shafiq

**Affiliations:** 1 School of Liberal Arts and Education, Shandong Xiehe University, Shandong, China; 2 Faculty of Foreign Languages, University of Foreign Languages and Information Technology, Ho Chi Minh City, Vietnam; 3 Department of Educational Policies, Faculty of Education, Umm Al-Qura University, Makkah, Saudi Arabia; 4 College of Computer and Information Sciences, Princess Nourah Bint Abdulrahman University, Riyadh, Saudi Arabia; 5 College of Computer and Information Sciences, Imam Mohammad Ibn Saud Islamic University (IMSIU), 11673 Riyadh, Saudi Arabia; 6 School of Information Engineering, Qujing Normal University, Yunnan, China

Correction to: *Scientific Reports* 10.1038/s41598-024-74406-4, published online 08 October 2024

In the original version of this Article Affiliation 5 was incorrectly given as ‘College of Computer and Information Sciences, Imam Muhammad Ibn Saud Islamic University (IMSIU), Riyadh, 11673, Saudi Arabia’. The correct affiliation is listed below.

College of Computer and Information Sciences, Imam Mohammad Ibn Saud Islamic University (IMSIU), Riyadh, 11673, Saudi Arabia.

The original Article has been corrected.

